# Haematological and CD4^+^ T cells reference ranges in healthy adult populations in Gojjam zones in Amhara region, Ethiopia

**DOI:** 10.1371/journal.pone.0181268

**Published:** 2017-07-19

**Authors:** Wondemagegn Mulu, Bayeh Abera, Zewdie Mekonnen, Yesuf Adem, Mulat Yimer, Yohannes Zenebe, Asmare Amuamuta, Wondimu Gebeyehu

**Affiliations:** 1 Department of Medical Microbiology, Immunology and Parasitology, College of Medicine and Health Sciences, Bahir Dar University, Bahir Dar, Ethiopia; 2 Biomedical Research Department, Biotechnology Research Institute, Bahir Dar University, Bahir Dar, Ethiopia; 3 Department of Biochemistry, College of Medicine and Health Sciences, Bahir Dar University, Bahir Dar, Ethiopia; 4 Amhara Region Health Research Laboratory Center, Bahir Dar, Bahir Dar, Ethiopia; Universiti Putra Malaysia, MALAYSIA

## Abstract

**Introduction:**

Establishing national population haematological and immunological reference ranges are essential for clinical management of patients. However, there is scarcity of information on community based haematological reference ranges established from Ethiopian population. Therefore, this study aimed at determining haematological and CD4^+^ T cells reference ranges in healthy adults from East and West Gojjam zones, Ethiopia.

**Methods:**

Community based cross-sectional study was conducted from May 2015 to December 2015 in healthy adult residents of Gojjam zone. A total of 481(246 females and 235 males) healthy adults enrolled in the study. Healthy adults were defined by medical history, physical examination and laboratory screening for HIV, HBV, HCV and intestinal parasitosis. Haematological parameters were measured using haematology analyzer MindrayBC320 (Mindray Biomedical electronic Corporation, China). CD4^+^Tcells were enumerated using FACS count (Becton Dickinson).

**Results:**

The median age of the participants was 25 years. The overall median and 95^th^ percentile of CD4^+^ T cells count were 869 cells/mm^3^ and396–1598 cells/mm^3^, respectively. Females had a significantly higher CD4^+^ T cell counts compared to males (P = 0.002). The 95^th^ percentile range for red blood cells (RBCs) was 3.93–6.1 x 10^6^cells/mm^3^and for hematocrit (Hct) was 40–58% while for hemoglobin (Hb) was 15.69–17.84g/dl. Males had significantly higher values of RBC and Hct than females (P < 0.001). Females (120–379 x 10^6^ cells/mm^3^) had significantly higher platelet counts than males (106–352 x10^6^ cells/mm^3^) (P < 0.001). The overall median of WBC was6.78 x10^3^/mm^3^and its95^th^percentile range was3.5–11.5 x10^3^/mm^3^. The overall 95^th^ percentile range of MCV, MCH and MCHC were 89.5–107.5 fl, 28–34 pg and 30–33.2g/dl, respectively. The higher mean absolute count of RBCs was found in the youngest age groups (P = 0.03). The mean count of RBCs and Hct were significantly higher in highschool completed and above than other participants (P < 0.001). The lower and upper limit of platelet counts was significantly higher in highland (118 -383x10^6^ cells/mm3) compared to lowland residents (107–352 x10^6^ cells/mm3) (P < 0.001). Moreover, it was significantly higher in residents with better monthly income (124–383 x10^6^ cells/mm3) compared to the counters (115–368 x10^6^ cells/mm^3^) (P = 0.02).

**Conclusions:**

Some of the haematological and CD4^+^ T cells reference ranges of the healthy adults in this study showed variations with the reference ranges used and reported so far in Ethiopia, Africa and Western countries. We recommend further study considering gender, altitude, and residency in other parts of Ethiopia to establish national reference ranges for Ethiopian population.

## Introduction

Health and disease can only be distinguished by accurate and reliable reference ranges of a particular test [1]. Haematological and CD4^+^ T cells reference ranges are useful both in the clinical and research areas [2]. They are primarily used for accurate interpretation of laboratory results, identifying abnormal laboratory results, guiding patient diagnosis and clinical managements [2]. Reference ranges are essential to screen physiological or pathological conditions and monitor patho-physiological changes after infection or disease states, or following the administration of drugs in therapeutic or clinical interventions and vaccine studies [3].

Haematological tests are used to screen the presence and type of blood disorders such as anemia and leukemia as well as different infections, immune status and disease progression [1,4]. Particularly with the HIV pandemicity and its very serious effect on sub-Saharan Africa, local community based haematological and CD4^+^ T—cell count for healthy populations is critical in supporting decisions of treatment initiation, monitoring disease progression and response to antiretroviral therapy [5].

Though haematological laboratory reference ranges are an important tool for identifying abnormal laboratory results, they vary due to differences in age, sex, lifestyle, ethnic background, climate and altitude[6,7].Furthermore, numerous studies in African, Asian and Western countries reported that reference ranges vary considerably in different populations, within population, groups, geographical regions, climate, race and dietary habits [3, 7–11]. Likewise, the normal ranges of red blood cell counts (RBCs), haemoglobin (Hb) concentration, hematocrit (Hct), mean cell volume (MCV), total white cell count (WBC), platelet and CD4^+^ T- cell counts are known to vary with age, sex, dietary patterns, ethnic origin, genetic and environmental factors [6, 7].

Few studies conducted in Ethiopia among apparently healthy individuals attending health institutions reported the presence of significant differences in normal laboratory ranges compared with those of Western countries [1, 5]. Thus, the establishment of haematological reference ranges specific for Ethiopian population has particular importance for interpretation of laboratory test results and provision of quality services in the health care delivery.

Differences have been documented between reference ranges of western countries, African populations and between different African ethnic groups. According to Clinical Laboratory Standard Guidelines (CLSI), each laboratory should establish their own reference ranges from the local population or validate those obtained from different settings. However, clinicians and researchers in Ethiopia are still using haematological reference ranges of western countries without any validation mechanism. Therefore, lack of own reference ranges may result in inappropriate management of patients and other decision making. Moreover, the precise absolute CD4^+^ T cell counts are important surrogate marker to determine the appropriate staging and treatment of HIV infected individuals. Therefore, there is atop urgent to have a base line data of reference ranges of haematological indices for healthy population at the community level. Thus, this study aimed at determining the reference ranges of haematological and CD4^+^ T cells indices in the community of Gojjam Zones, Ethiopia.

## Materials and methods

### Study design, period and setting

Community based cross-sectional study was conductedfrom May 2015 to December 2015 among healthy adult residents in Jabitehnan and DebreMarkos administrative districts of Gojjam, Amhara National Regional State. Jabitehnan district is a lowland area located in West Gojjam Zone 160 Kilometers away from the regional city. It has a total population of 287,045. DebreMarkos is the capital city of East Gojjam zone located 268 Kilometers away from Bahir Dar city. It has an altitude of 1800m above sea level with a total population of 93,900 [12]. The collected blood samples from each district processed and analyzed at DebreMarkos Referral hospital laboratory. The source populations were all healthy adults of the East and West Gojjam Zones in the Amhara National Regional State. Healthy adults with age ≥ 18 years of both sexes from the selected households in the study area were the study population.

### Sample size and sampling

We calculated the sample size using single population proportion formula. It was determined by taking a proportion of 0.5 assuming that, mean (μ) population for each parameters is known to be 50%, 2% marginal error, 95% confidence intervals. Therefore, a sample size of 481 was determined. Considering altitude difference, one lowland and one highland districts taken randomly from Gojjam administrative zones. The number of sub-districts were determined proportional to their population size of each districts. Sub-districts selected using simple random sampling technique. Moreover, from the selected sub-districts, the number of households were determined proportional to their population size. We selected n^th^ households from each districts using systematic random sampling after getting the n^th^ value by dividing the total number of households with the total number of selected households. From each selected households, a healthy adult (≥18 years old) was selected using lottery method. Thus, only one adult from each household included in the study.

### Variables

Reference ranges of haematological parameters and CD4^+^T cells were dependent variables while age, sex, altitude, residence, monthly income, educational and marital status were independent variables.

### Inclusion criteria

Healthy adults of permanent residents of the study area with age **≥** 18 years of both sexes from the selected households.

### Exclusion criteria

Age <18 years, adults with intestinal parasitic infections, haemoparsites, skin rashes, history of blood transfusion < 6 months, HIV positive, HBsAg positive, HCV positive, pregnant, on any medication, exhibiting febrile symptoms, observable mental illness, disabled, smokers, chronic alcohol drunkers, anaemic, chronic diseases and acutely ill patients as per the recommendations of WHO excluded from the study. Blood samples were therefore obtained from healthy adults selected to generate haematological values.

### Operational definitions

**Healthy adults:** the absence of disease or disabilities based on medical history, physical diagnosis, clinical sign and symptom plus laboratory investigations.

**Reference range:** the range between, and including two reference values defined by a specific percentage (usually 95%) for haemato-immunological parameters of healthy individuals.

**95**^**th**^ **percentile ranges:** It is ranges between, and including the 2.5^th^ percentile and the 97.5^th^ percentile.

### Data collection

Initially, all participants screened with medical history and physical examination by physicians. Apparently healthy adults further screened for intestinal parasitosis, diabetes, anaemia, HIV, HBsAg and anti- HCV. Socio-demographic factors collected from each participant using a structured questionnaire via face to face interview.

### Laboratory analysis

After completion of the interview, 5 ml of venous blood collected from each participant in the morning and stored in vacutainer tube containing ethylenediamine tetra acetic acid (EDTA).Haematological parameters such as WBC count, differential white cell count (including neutrophilis, mixed white cells and lymphocytes), RBC count, Hb, Hct, MCV, MCH, MCHC, and platelet count performed using an automated haematology analyzer MindrayBC320(Mindray Biomedical Electronic Corporation limited, China).As per the manufacturers instruction the machine aspirated 13μl of blood into a chamber and diluted with a balanced isotonic solution. The diluted blood sample was split in to two parts: one went for RBC and platelet counting and the other for total WBC, Hb and differential counting.

Fluorescence-activated cell sorter (FACS) system (Becton Dickinson, USA) was used to enumerate absolute values for CD4^+^ T cells. FACs count system uses single reagent tube for enumeration of absolute CD4 ^+^ T cells count in whole blood. In brief, 50 μl of whole blood mixed and incubated at 21^o^c for 20 minutes with 20 μl of a CD4 reagent. Red blood cells lysed by adding 450 μl of fluorescence-activated cell sorter lysing solution (Becton Dickinson Immunocytometry Systems). The tubes incubated at 21^o^c for 10 minutes, and then analyzed with the FACS Counts’ Cell Quest software (Becton Dickinson Immunocytometry Systems) within six hours.

### Quality control

By using quality control (Multicheck Becton Dickinson Immunocytometry Systems), the accuracy of the technique assessed. For CD4 ^+^ T cells BDFACs Count ^TM^ controls for high, medium, low and zero controls were used during enumeration to validate CD4 machine. Moreover, for haematological parameters, Mindray BC320 calibrator and low, medium and high control samples were run during each day of analysis as defined in the instrument by the manufacturer’s manual.

### Data analysis

Data was entered and analyzed using Statistical Package for Social Science (IBM Corp- Released 2011. IBM SPSS statistic Armonk, NY: IBM Corp). Descriptive statistics used to determine the mean, median and 95^th^ reference range of each parameter. Chi-square, independent sample T-test and one-way ANOVAs employed to see the association between variables. All statistical tests were two tailed, and P.value< 0.05 considered statistical significant.

### Ethical considerations

Ethical clearance was obtained from the research ethics review committee of Biotechnology Research Institute of Bahir Dar University. Written consent was obtained from each study participants to provide clinical specimen for screening and research purpose. The Amhara Regional State Health Bureau provided permission to collect blood sample from Gojjam zone. Therefore, the institutional research ethics review committee of the Amhara Regional State Health Bureau, Research and Technology Transfer Core Process approved ethical clearance to haematological parameters on blood samples. All participants diagnosed for any illness were treated accordingly. Information obtained at any course of the study was kept confidential.

## Results

### Participants’ characteristics

Table 1 depicts the characteristics of participants. A total of 481(246 females and 235 males) community healthy adults enrolled in the study. The age range was 18–65 years (Mean = 29.9). Two hundred eighty (58.2%) were highschool completed and above. The majority (97.1%) were urban residents. Most (84.2%) of the participants were from highland locations.

**Table 1:** Demographic characteristics of study participants.

Variables	Frequency	Percent
**Sex**		
Male	235	48.9%
Female	246	51.1%
**Age (years)**		
18–25	257	53.5
26–35	119	24.7
36–45	60	12.5
46–55	27	5.6
56–65	18	3.7
**Residency**		
Urban	467	97.1
Rural	14	2.9
**Educational status**		
Highschool completed and above	280	58.2
Illiterate & elementary completed	201	41.8
**Income**		
≤1500 ETB	415	86.3
>500 ETB	66	13.7
**Altitude**		
Highland	405	84.2
Lowland	76	15.8
**Marital status**		
Single	245	50.9
Married	236	49.1

ETB: Ethiopian Birr

### Haematological parameters and CD4^+^ T cells

As shown in Table 2, the overall95^th^ percentile range of absolute CD4count was 396–1598 cells/mm^3^. The mean and 95^th^ percentile range of absolute CD4^+^ T cells count for menwere854 cells/mm^3^ and414-1474 cells/mm^3^, respectively. For women, it was 1000 cells/mm^3^ and 436–1695 cells/mm^3^, respectively. Females had a significantly higher CD4^+^ T cells count than males (P = 0.002).

**Table 2:** Mean, median and 95% reference ranges values of haematological and CD4 ^+^ T cells in relation to sex and age profile of healthy adults.

Variables	Parameters											
	WBC(10^3^/mm3)	RBC(10^6^/mm3)	Platelet (10^6^/mm3)	Lympho Cyte %	Neutrophil %	MID %	HB(g/dl)	Hct%	MCV (FL)	MCH (pg)	MCHC (g/dl)	CD4(cells/mm3)
**Sex**												
**Female**												
Mean	6.87	4.69	235	33.4	55.1	11.8	17.4	46.4	98.7	31	31.5	1000
Median	6.6	4.7	227	32	55	10	14.9	46.1	98.7	31	31.5	974
95% range	3.6–11.9	3.9–5.8	120–379	18–54	34.5–71.8	6.1–26.3	12–18	39–55	90.3–106	28–34	30–33.2	436–1695
**Male**												
Mean	6.69	5.29	212	31.9	56.1	11.9	17.17	52	98.9	31.4	31.7	854
Median	6.65	5.34	208	31.1	57	11	16.8	52	99	31	32	800
95% range	3.5–10.9	3.9–6.2	106–352	15.1–53.4	35–73.8	6.8–21	13.6–19.8	45–59	90–109	28–34.4	30–33.3	414–1474
**P-value**	**0.4**	**0.000**	**0.000**	**0.11**	**0.29**	**0.94**	**0.87**	**0.000**	**0.31**	**0.03**	**0.007**	**0.002**
**Age (years)**												
**18–25**												
Mean	6.94	5.1	222	31.8	56.7	11.28	15.86	49.87	98.3	31.1	31.6	938
Median	6.7	5.1	218	30	58.2	10.4	16	50	98.5	31	31.7	863
**26–35**												
Mean	6.7	4.9	227	33.1	55.2	12.4	20	48.98	98.9	31.3	31.7	990
Median	6.6	4.9	219	33	55.8	11	15.9	49	99	31.1	32	922
**36–45**												
Mean	6.2	4.9	222.9	35	52.8	11.9	19.54	47.8	99.9	31.5	31.7	935
Median	6.1	4.8	212	35	53	10.8	15	47	100	31.5	32	858
**46–55**												
Mean	7.1	4.8	228	32.9	54.1	12.97	14.9	47.5	99.7	31.1	31.4	876
Median	7.1	4.7	227	30	55.5	13	15	46	100.5	31	31.5	808
**56–65**												
Mean	6.7	4.9	205	32.9	51.2	15.9	15.18	48.1	99.1	31	8.5	831
Median	6.6	4.9	212.5	31.5	46.7	13.35	15.15	48.3	99.1	31	8	822
**P-value**	**0.28**	**0.03**	**0.83**	**0.22**	**0.04**	**0.08**	**0.23**	**0.03**	**0.02**	**0.09**	**0.61**	**0.82**
**Male and female combined**												
Mean	6.78	**4.69**	**223.5**	**32.6**	**55.6**	**11.85**	**15.6**	**49.2**	**98.8**	**31.2**	**31.6**	**932**
Median	6.6	**4.98**	**218**	**32**	**56.4**	**11.0**	**15.9**	**49**	**99**	**31.0**	**31.8**	**869**
95% range	**3.5–11.5**	**3.93–6.1**	**113.9–372**	**17.9–53.4**	**34.3–72.1**	**6.0–23**	**12–19**	**40–58.2**	**89.5–107.5**	**28–34**	**30–33.2**	**396–1598**

MID: Basophil + Eosinophil+ Monocytes

The overall (male and female combined) median and 95^th^percentile range of RBCs were 4.69 x10^6^cells/mm^3^ and 3.93–6.1 x10^6^cells/mm^3^, respectively and for Hct were 49.2% and 40–58.2%, respectively. Moreover, the overall median and 95^th^ percentile of Hb and platelets were 15.9g/dl and 15.69–17.84g/dl, respectively. The overall median of platelets was 218x10^6^ cells/ mm^3^and its95^th^ percentile was113.9–372 x10^6^cells/mm^3^. The95^th^ percentile ranges of RBC and Hct for men were3.9–6.2x10^6^ cells/mm^3^ and 45–59%, respectively. The corresponding values for women were 3.9–5.8x10^6^ cells/mm^3^ and 39–55%, respectively. The overall 95^th^ percentile range for WBC was 3.5–11.5x10^3^/mm^3^and for neutrophil was 34.3–72.1%(Table 2).

The overall 95^th^ percentile range of MCV, MCH and MCHC were 89.5–107.5 fl, 28–34pg and 30–33.2g/dl, respectively. The 95^th^ percentile range of MCH and MCHC for men were28-34.4pg and 30–33.3g/dl, respectively. The corresponding values for women were 28–34 pg and30–33.2g/dl, respectively (Table 2).

Higher mean absolute count of RBCs was observed in the youngest age groups compared to olders (P = 0.03). Males had significantly higher values of Hct (45–59%) than females (39–55%) (P = 0.002). Females had a significantly higher lower and upper platelet counts (120–379 x10^6^cells/mm^3^) against 106–352 x10 ^6^ cells/mm^3^ for males (P < 0.001) (Table 2). The Mean absolute counts of RBCs (P < 0.001) and values of MCHC (P = 0.002) were significantly lower in females than males. The mean absolute count of neutrophil (P = 0.04) and values of Hct (P = 0.03) were significantly lower in older compared to younger age groups. However, the mean absolute value of MCV was significantly lower in younger compared to older age groups (P = 0.02) (Table 2).

Mean absolute count of RBCs and values of Hct were significantly higher in highschool completed and above compared to other participants (P<0.001) (Table 3). Both the lower and upper limit of platelets count was significantly lower in lowland than highland residents (P<0.001). Table 4 shows haematological reference ranges of highland and lowland residents.

**Table 3:** Mean, median and 95% reference range values of haematological and CD4^+^ T cells in relation to educational status of healthy adults.

Educational status	High school completed and above			Illiterate & elementary completed			
Parameters	Median	Mean	95% range	Median	Mean	95% range	P.value
CD4^+^ (cells/mm3	8.59	9.43	425–1597	8.95	9.41	414–1565	0.97
Lymphocyte %	31.3	32.38	16.6–52.8	32	33	19–55	0.48
WBC (10^3^/mm^3^)	6.6	6.7	3.8–11.6	6.6	6.87	3.7–11	0.42
Neutrophils %	57.2	56.17	34.5–72.8	55	54.79	36.8–71	0.17
MID (%)	10.4	11.23	6–22	11.00	12.68	7–23	0.01
RBC (10^6^/ mm^3^)	5.1	5.08	4.1–6.1	4.79	4.88	4.1–6.1	0.000
Hb (g/dl)	16.00	16.77	12–19	15.00	18.04	12–18.7	0.41
Hct (%)	50	50	40–59	47.6	48	40–57.6	0.000
MCV (Fl)	99	98.4	90–107	100	99.4	92–107	0.16
MCH(pg)	31	31.1	28–34	31.2	31.3	28–34	0.1
MCHC (g/dl)	31.7	31.6	30–33.6	32	31.6	30–33	0.78
Platelet (10^6^/mm^3^)	219	226	115–383	217	220	119–365	0.34

MID: Eosinophil+ basophile+ monocytes

**Table 4:** Mean, median and 95% range values of haematological and CD4^+^ T cells in healthy adults in relation to altitude.

Altitude	Highland			Lowland			
Parameters	Median	Mean	95% range	median	Mean	95% range	P.value
CD4^+^ (cells/mm3)	8.84	9.65	425–1551	8.16	8.19	415–1565	0.06
Lymphocyte %	32	32.9	18–52.8	29	31.1	20–54	0.14
WBC (10^3^/mm^3^)	6.6	6.78	3.6–10.9	6.6	6.81	3.7–12.3	0.92
Neutrophils %	56.6	55.5	34.5–72.8	56	56.1	35–72	0.66
MID %	10.4	11.7	6.0–23.3	12.00	12.66	7–21	0.23
RBC (10^6^/ mm^3^)	5.00	5.01	4.1–6.1	4.87	4.92	4–6	0.26
Hb (g/dl)	16	17.65	12.5–18.9	15	15.34	12–19	0.28
Hct %	49	49.2	40.5–58	48	49.1	40–59	0.81
MCV (Fl)	99	98.6	90–107	100	99.7	90–107	0.81
MCH(pg)	31	31.18	28–34	31	31.27	28–34	0.67
MCHC (g/dl)	31.9	31.66	30–33.3	31	31.42	30–33	0.05
Platelet (10^6^/mm^3^)	223	229	118–383	187	191	107–352	0.000

The mean absolute count of platelets (P = 0.02), RBCs (P = 0.004) and values of Hct (P = 0.03) were significantly higher in residents with better monthly income compared to the counters. Table 5 shows haematological reference ranges of high and low income residents.

**Table 5:** Mean, median and 95% reference range values of haematological and CD4+ T cells in relation to monthly income in healthy adults.

Monthly income	≤ 1500 ETB			>1500 ETB			
Parameters	Median	Mean	95% range	Median	Mean	95% range	P.value
CD4^+^ (cells/mm3)	8.69	9.26	414–1551	8.68	1.04	417–1597	0.17
Lymphocyte %	31.2	32.36	18–54	33.9	34.19	20.3–53.4	0.15
WBC (10^3^/mm3)	6.6	6.83	3.7–11.6	6.6	6.49	3.5–11	0.23
Neutrophils %	56.8	55.76	34.3–72.8	56.00	54.76	37–71	0.43
MID %	11	11.99	6–23	10.00	11.00	6–23.8	0.23
RBC (10^6^/ mm^3^)	4.95	4.97	4.0–6.1	5.23	5.19	4.1–6.1	0.004
Hb (g/dl)	15.7	17.5	12–18.8	16	16.1	12–18.9	0.52
Hct %	49	48.98	40–58	50.5	50.57	39.9–58	0.03
MCV (Fl)	99	98.9	90–107.6	97.8	97.7	90–107	0.54
MCH(pg)	31	31.23	28–34	31	30.93	28.4–33.4	0.15
MCHC (g/dl)	31.7	31.6	30–33.2	32	31.7	30–33.6	0.41
Platelet (10^6^/mm^3^)	216	220	115–368	232	241	124–383	0.02

## Discussion

In Ethiopia diagnostic and treatment health facilities employed haematological reference ranges adopted from western countries. At the same time there is no uniform usage of a hematological parameters reference ranges at the national level. Health care providers may obtain these reference ranges from pocket and text books, WHO reports, browsing internet and inserts from kits to compare the reported values. A variety of inserted kits are also available from several companies as a result of different health facilities uses various types of automated machines such as Mindray, Cell-DYn 1800, Sysmex (Xs-500i) and Cell-DYN Rubey. In spite of these, limited studies on haematological reference ranges have been reported among apparently healthy individuals attending hospitals either for blood donation, HIV counseling and testing [5, 14]. Therefore, this study presents community based haematological reference ranges in healthy adults of Ethiopia and may be a baseline for Federal Ministry of Health for future national reference range establishment.

The mean absoluteCD4^+^ T cell counts in this study is not different from the reference ranges used in the study setting during the study period[13]. It is higher than previous studies in Ethiopia[1,5,14] and other African countries[4, 6]. Moreover, the lower and upper limit ofCD4^+^ T cells reference range in this study was not in agreement with the reference ranges approved for the national ART Guideline [13]. Furthermore, the reference range of females’ absolute CD4^+^ T cell counts in this study is higher than studies in Southern Tanzania [3], and Central Republic of Africa [15], while lower than a study done in Turkey [16] and similar with studies conducted in Kenya [7], and Botswana [8]. The reference ranges of males CD4^+^T cell counts in this study varies with the reference ranges of previous studies in Ethiopia [5], other African countries [4, 7, 8, 15] and Turkey [16]. This could be due to variations in study population, ethnicity, genetic, diet, geographical and environmental factors [6, 7]. The significantly higher mean and lower and upper limit of CD4^+^ T cell counts in females than males is comparable with earlier studies in Ethiopia [1,5,14] and Tanzania [4]. This could be due to the effect of sex hormones as peripheral lymphocytes have receptors to estrogen and androgen. The higher lower and upper limit of CD4^+^ T cells among highland compared to lowland residents in the present study might be associated with the effect of erythropoietin release following lower oxygen tension in highland areas cross-stimulating lymphoid progenitor cells. Therefore, federal ministry of health should update it with respect to gender and altitude based on the present and past reports of Ethiopia.

In the present study, the lower and upper limit reference ranges of RBCs and Hct was higher than previous studies in Bahir Dar, Ethiopia [14], and Ghana [6]. The lower and upper reference ranges of males RBCs in this study is lower than reference range of males in Zimbabwe[17], South Tanzania [3], Botswana[8], and Wintrobes standard [18], but higher than other African countries [6, 7,19, 20]. On the other hand, the lower and upper reference ranges of females RBC and Hb in this study was higher than findings reported in Southern Tanzania [3], Ghana [6], Kenya [7], Botswana [8], and Rural Uganda[19]. This might be due to variation in ethnicity, altitude and dietary habits. Moreover, the lower and upper limit of RBC significantly higher in males than females and in younger than older age groups.

In this study both the lower and upper limit of Hct is higher than reference ranges reported in African countries and used in the study community [Table 6]. The lower and upper limit Hct reference range of males and females in this study was higher than previous studies in Ethiopia [15], other African countries [6,7, 17, 19, 20], Wintrobes standard and currently used ranges in the study area [Table 6]. Moreover, the lower and upper limit reference ranges of Hct in both males and females are higher than Wintrobes standards [18], and currently used ranges in the study area [Table 6]. Therefore, federal ministry of health should consider such variations.

**Table 6:** Comparison of reference ranges values of the present study with other studies in Ethiopia, African and Western countries, 2016.

Parameters	Present study			African countries (3–4)			Ethiopia (1, 5, 14, 23)			Western countries (10, 11, 16)			Currently used (13)			Wintrobe Standard (18)	
	Male	Female	Total	Male	Female	Both	Male	Female	Both	Male	Female	Both	Male	Female	Total	Male	Female
CD4^+^ (Cells/mm^3^)	414–1474	436–1695	396–1598	342.8–1435.3	403–1498.8	361.8–1473.7	324.7–1244.7	415.3–1370.7	351–1269.3			449.3–1680.5	-	-	500–1300 cell/mm^3^		
WBC (10^3^/mm^3^)	3.5–10.9	3.6–11.9	3.5–11.5	3.16–7.65	3.48–7.98	3.04–9.08	3–10	3.1–10.9	3.1–9.7				-	-	4–11 4.3–10 4.3–10		
RBC (10^6^/mm^3^)	3.9–6.2	3.9–5.8	3.93–6.1	4.35–6.12	3.72–5.51	3.83–6.18	3.46–6.18	3.24–5.56	2.95–5.8	3.77–5.5	3.58–5.0		4.2–6	3.6–5.6	3.8–5.1	4.5–6.3	4.2–5.5
Platelet (10^6^/mm3)	106–352	120–379	113.9–372	141.6–357.2	154.8–401.4	114–388.3	84–386	94.4–404.8	101.7–428.3	122–397	142–377.5		140–440	140–440	140–440	150–450	150–450
HB (g/dl)	13.6–19.8	12–18	12–19	12.6–16.7	10.1–14.5	11.2–17.2	11.4–18	10.6–16.4	9.95–17.55	12.8–16.7	11.64–14.83		12–18	12–18	12–18	14–18	12–16
HCT %	45–59	39–55	40–58.2	35.5–49.8	30.3–44.3	30.6–49.6	34.5–54.9	31–50.6	28.45–51.7	37.4–50.5	34.8–47.7		34–54	34–54	34–54	41–51	37–47
MCV (fl)	90–109	90.3–106	89.5–107.5	75.5–96.4	73.3–95.1	69.7–97.6	68.45–100.2	69.1–98.95	69.8–99.9	NA	NA		81–103	81–103	81–103	NA	NA
MCH (pg)	28–34.4	28–34	28–34	23.9–32.3	24.7–32.1	22.7–33.2	22.5–34.6	21.2–35.5	19.75–36.2	NA	NA		27–32	27–32	27–32	NA	NA
MCHC (g/dl)	30–33.3	30–33.2	30–33.2	30.9–35.3	30.5–34.9	30.2–35.4	27.9–37.2	24.1–37.1	28.05–37.1	NA	NA		32–36	32–36	32–36	NA	NA

The median, lower and upper limit of Hb reference range in this study conforms to a study conducted in Ethiopia [14], and reference ranges used in the study hospital [Table 6]. However; it was higher than studies reported in Ghana [6], rural Uganda [19] and Iran [21]. In the present study the lower and upper limit of RBCs and Hct in males are significantly higher than females (P < 0.001). This conforms to earlier studies in Ethiopia [5, 15], African countries [6–8, 17, 20, 22], Gaza strip [9] and Wintrobes standard [18]. This could be due to loss of blood in menstrual cycles in females and the influence of differences in the hormone androgen between females and males on their erythropoiesis. Moreover, the median RBCs and Hct values were significantly higher among highschool completed and above compared to other participants (P < 0.001). The highest mean and median values of RBC were found in the youngest age groups. However, comparison was not made due to paucity of data reported before.

In this study, both the lower and upper limit of WBC reference ranges was higher than earlier studies in Ethiopia [1,14, 23] and African countries [3, 6,7, 22] while lower than reports from Western countries [10, 11, 16] and conforms to ranges used in the study community [Table 6]. Moreover, the lower and upper limit of WBC reference ranges of both males and females was higher than earlier studies in Africa [3, 6, 7] and Ethiopia [1, 14, 23]. Moreover, the overall median and reference ranges of neutrophil count of this study is higher than previous studies in Ethiopia [1, 5, 14], Southern Tanzania[3], and Zimbabwe[17], while similar with studies in Gondar[23], and multiethnic population in Malaysia[9].

In this study statistical significant difference on the reference intervals of neutrophilis between males and females was not found. Similar reports have been found in Bahir Dar, Ethiopia [14], Southern Tanzania [3], Botswana [7], and Zimbabwe [17]. However, in Nigeria [20], males had significantly higher neutrophilic interval than females.

The lower and upper limit of platelet reference ranges in this study is lower than ranges used in the study community [Table 6] and Wintrobes standard [18]. The overall median and upper limit of platelets reference range in this study is lower than previous studies in Ethiopia [1, 14] and other African countries [3, 6,7]. However, its lower limit is higher than the above studies [1, 3, 6, 7, 14]. Moreover, the median, lower and upper limits of male’s platelets reference ranges in the present study are lower than previous studies in Botswana [8], Zimbabwe [17], Gaza strip [9] and UK [10]. It is also significantly lower compared to reference ranges used in the study setting (Table 6) and Wintrobes standard [18]. Furthermore, the median and reference ranges of female platelets in this study also lower than previous studies in Southern Tanzania [3], Botswana [7], Zimbabwe [17], and Gaza strip [9]. Dietary, environmental and genetic factors might contribute for the lower platelet counts compared to Wintrobes standard and other countries.

In this study, both the lower and upper limits of platelets were significantly higher in females than males. This was in agreement with studies conducted in Ethiopia [1, 14] and Northern Tanzania [22]. Higher platelet counts in females compared to males might be due to the variations in hormone types and concentrations in the different sexes and the effect of erythropoietin release in response to regular menustration cross- stimulating megakaryopoiesis. Moreover, both the lower and upper limit of platelets was significantly higher among highland compared to lowland residents (P< 0.0001) and among those who had better compared to lower monthly income residents (P = 0.03). This might have a direct or indirect influence on the proliferation of megakaryocytes.

The lower and upper limit of MCV and MCH reference ranges in the present study is higher than earlier studies in Ethiopia [14, 23] and other African countries [3, 6, 7, 17] and ranges used in the study area during data collection [Table 6]. However, the reference range of MCHC in this study conforms to previous studies in Ethiopia [14], Southern Tanzania [3], Ghana [6], and Zimbabwe[17]. In this study the mean values of MCH and MCHC was significantly higher in males than females while the mean values of MCV was not significantly associated with gender difference. This was similar with previous studies in Botswana [8], and Zimbabwe [17]. However, significantly higher values of MCV in males than females reported in Ghana [6], and Zimbabwe [17]. Non-significant difference in MCH and MCHC values was also reported in Ghana [6], and Nigeria [20].

In this study the mean absolute count of RBCs, platelets and Hct significantly higher among residents with better monthly income compared to others (P = 0.03). This might be due to the influence of diet on the process of blood cell formation. However, due to lack of data comparison was unable to make.

## Conclusions

Some of the haematological and CD4^+^T cells reference ranges of healthy adults in Gojjam showed variations with reference ranges used in the study community, reported in Africa, Western countries and Wintrobes standard. Difference in altitude and gender significantly affects the reference intervals. Therefore, considering gender, altitude and residency further studies in other parts of Ethiopia are recommended to establish reference intervals for Ethiopian population.
